# The Transformative Role of Nanotechnology in the Management of Diabetes Mellitus: Insights from Current Research

**DOI:** 10.3390/biom15050653

**Published:** 2025-05-01

**Authors:** Natalia G. Vallianou, Maria Dalamaga, Argyro Pavlou, Eleni Rebelos, Nikolaos Nektarios Karamanolis, Eleftheria Papachristoforou, Evangelos Mavrothalassitis, Ioanna Eleftheriadou, Nikolaos Tentolouris, Dimitris Kounatidis

**Affiliations:** 1 First Department of Internal Medicine, Sismanogleio General Hospital, 15126 Athens, Greece; argirpavlou@gmail.com (A.P.); evangethalassitis@gmail.com (E.M.); 2 Department of Biological Chemistry, Medical School, National and Kapodistrian University of Athens, 11527 Athens, Greece; madalamaga@med.uoa.gr; 3 Diabetes Center, First Department of Propaedeutic Internal Medicine, Medical School, National and Kapodistrian University of Athens, Laiko General Hospital, 11527 Athens, Greece; eleni.rebelos@utu.fi (E.R.); lfmedpap@gmail.com (E.P.); ntentol@med.uoa.gr (N.T.); dimitriskounatidis82@outlook.com (D.K.); 4 Second Department of Internal Medicine, Medical School, National and Kapodistrian University of Athens, Hippokration General Hospital, 11527 Athens, Greece; inektkaramanolis@gmail.com

**Keywords:** diabetes mellitus, diabetic wounds, insulin delivery systems, nanomedicine, nanotechnology, phytochemicals

## Abstract

Nanotechnology refers to the science that modulates molecules to the nanoscale dimension. Nanomedicine, i.e., the utilization of nanotechnology for diagnosing and treating several disorders, is a subject of ongoing research. The concept behind nanomedicine in diabetes mellitus (DM) treatment stems from the need to ameliorate absorption and distribution of antidiabetic therapies in order to overcome barriers, namely the pH throughout the gastrointestinal tract, the gut microbiota, the temperature/heat and the difficulties in the incorporation of drugs into the cells. Thus, the scope of nanomedicine is particularly challenging and demanding, considering the fact that the human body is a perpetually changing entity in order to achieve homeostasis. In this review, we will delve into various nanoparticles that are being studied in terms of antidiabetic treatment, their pros and cons and the expanding knowledge in this field. Despite the fact that nanomedicine seems to be very promising, there are still many gaps in our understanding of how the human body addresses its utilization. Moreover, its high costs, along with an as-yet unclear safety profile, remain a significant barrier to widespread adoption. In this review, we will describe both phytochemicals and chemical compounds that nanomedicine seeks to exploit in order to pave the way for a more efficacious and comprehensive management of diabetes mellitus.

## 1. Introduction

Diabetes mellitus (DM) is a chronic metabolic disorder characterized by insufficient production of insulin by the pancreas or by reduced ability to effectively utilize insulin in the peripheral tissues, thus resulting in hyperglycemia. According to the World Health Organization (WHO), in 2022, 830 million people had DM, an increase from 7% to 14% between 1990 and 2022 [1,2]. In addition, in 2022, approximately 59% of the adults living with DM did not receive any antidiabetic treatment [1,2]. Since DM is associated with major macrovascular and microvascular complications that impact both mortality and morbidity, there is an urgent need for accurate diagnosis and timely treatment.

Nowadays, effective antidiabetic treatment is often hindered by the relatively low bioavailability of glucose-lowering medications. Limitations related to the pharmacokinetics and pharmacodynamics of various antidiabetic regimens, particularly the uncontrollable release of the drug, may reduce patient compliance [3]. In addition, challenges associated with conventional antidiabetic treatments, such as varying pH levels throughout the gastrointestinal (GI) tract and enzymatic drug degradation, need to be addressed [4]. Therefore, more effective ways to overcome these drawbacks should be pursued. In this context, the advent of nanotechnology is highly appreciated.

Nanotechnology refers to the science that modulates atoms and molecules to minimize them at a nanoscale level, i.e., with at least one diameter of between 1 nm and 100 nm (nanometers), approximately [3,4,5,6]. Nanotechnology has multiple applications in various sciences, such as physics, chemistry, molecular biology, engineering and medicine [3,4,5].

The purpose of this review is to elaborate upon the several aspects of nanotechnology in the treatment of type 1 diabetes mellitus (T1DM), as well as type 2 diabetes mellitus (T2DM). We aim to further describe the reasons behind the perpetually evolving use of nanotechnology regarding anti-diabetic treatment. In addition, we will delve into the therapeutic advances of antidiabetic drugs at the nanoscale level and the future perspectives of nanotechnology in terms of treatment of T1DM and T2DM. In this review, the pros and cons of nanotechnology will be thoroughly described.

### Literature Search

For our manuscript, we searched the Pubmed database using the phrase “nanotechnology and diabetes drugs” and found 357 manuscripts during the past 5 years. Among these 357 publications, we have excluded manuscripts that dealt with (1) anti-inflammatory and antioxidant properties and not merely anti-diabetic features; (2) other metabolic disorders, such as MASLD and obesity; (3) cancer; (4) other diseases, such as Alzheimer’s disease, COVID-19, arthritis and glomerulonephritis; (5) diagnostics, i.e., regarding the utility of nanotechnology in diagnosing and monitoring diabetes; (6) manuscripts written in foreign languages. Thus, after the exclusion of 147 manuscripts, we included 210 publications. This review was based on the aforementioned publications. However, we acknowledge that all these articles cannot be covered in the context of this review.

## 2. Nanotechnology in Medicine

Nanomedicine is the field of nanotechnology occupied with the applications of nanoscale technologies in the diagnosis and treatment of various diseases [3,4,5,6]. Nanomedicine has made remarkable advancements, especially in the diagnosis and treatment of cancer [3,4,5,6]. It aims to improve the absorption of a variety of drugs as well as their release in a more controllable manner, while ensuring their safety [7,8,9,10,11,12]. Regarding T1DM and T2DM, we will refer to antidiabetic treatment options together with future advances that could be incorporated in DM treatment.

The concept behind nanomedicine in DM therapy stems from the need to ameliorate absorption and distribution of antidiabetic therapies in order to overcome several obstacles. Physical barriers, namely the pH throughout the GI tract, the gut microbiota, the temperature/heat and the difficulties in the incorporation of drugs into the cells, are among the major challenges. This task of nanomedicine could be particularly demanding, considering the fact that the human body is a perpetually changing entity in order to achieve homeostasis. However, applying nanotechnology in antidiabetic therapy could be very constructive and fruitful. Indeed, nanoparticles (NPs), i.e., particles ranging 1–100 nm in size, are loading forms that can carry a drug and improve its distribution throughout the body as well as its incorporation into the cell [7,8,9,10,11,12].

NPs used in DM treatment are spherical systems mainly categorized into the following four classes according to their physical and biological features: polymeric NPs or nanospheres, polymeric nanocapsules, liposomes and lipid NPs. Polymeric NPs are made of natural polymers, such as polysaccharides (chitosan, hyaluronic acid and sodium alginate) and proteins (gelatin, albumin) or synthetic polymers, namely polylactic acid (PLA) or poly lactic-co-glycolic acid (PLGA) [8,9,10]. Polymeric NPs consist of a polymeric matrix in which the drug is homogeneously dispersed, which is responsible for their protection from physical barriers and their controlled release. These polymeric NPs, also known as nanospheres, are capable of releasing both hydrophilic and lipophilic drugs in a controllable manner [9,12,13,14]. Polymeric nanocapsules differ from polymeric nanospheres because the solid surface polymer encompasses an oily core, where the drug is mainly dissolved. Polymeric nanocapsules also possess the ability to carry hydrophilic and lipophilic drugs, protect them from the host’s environment and improve their bioavailability [14,15,16,17,18]. The third class consists of liposomes. Liposomes are spherical structures made of one or more bilayers of phospholipids, which surround an aqueous phase. Their structure, which resembles the cytoplasmic cell membrane, accounts for their ability to deliver the drug to the exact lesions in the host. Therefore, these vehicles may allow for maximum tolerated dosing with fewer adverse effects [9,19]. The last category of NPs consists of lipid NPs, which differ from liposomes as they possess only one phospholipid layer that surrounds a core containing inverted micelles. Lipid NPs can be surrounded by surfactants and are capable of encapsulating small molecules, nucleic acids and even monoclonal antibodies [9,20].

**Figure 1:**
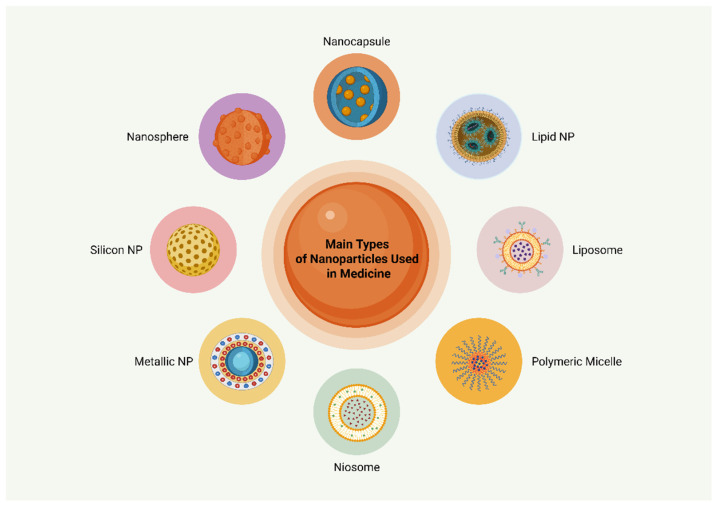
Main types of nanoparticles used in experimental medicine. Abbreviations: NP: nanoparticle. Created in BioRender. Kounatidis, D. (2025) https://BioRender.com/c5u8lm1. Assessed on 18 April 2025.

However, there are other classifications of NPs as well. In particular, NPs have also been classified as liposomes, nanospheres, polymeric micelles, solid lipids, metallic NPs, niosomes, and porous silicon NPs [3]. Niosomes are very similar to liposomes, but carry a non-ionic surfactant arranged in bilayers and stabilized by cholesterol. Due to their non-ionic nature, niosomes exhibit a very low toxicity rate and increased bioavailability [3,21]. In addition, metallic NPs are made of a metal structure, mainly gold, silver, zinc or iron oxide. Their major advantage is their ability to reach the targeted area with enhanced accuracy [20]. Nevertheless, as the field of nanomedicine is exponentially growing, it is expected that a variety of NPs will be discovered and exploited in the near future. Therefore, the classification of NPs seems to be constantly changing. Figure 1 illustrates the primary classes of NPs utilized in medicine.

## 3. Antidiabetic Therapies and Nanotechnology

### 3.1. Insulin Therapy

Nanotechnology in the treatment of DM has mainly focused on the delivery systems for insulin. More specifically, the subcutaneous route of insulin administration, which is currently the main treatment modality for T1DM, has several drawbacks. The need for repeated injections daily may lead to patients’ discomfort and often limited compliance and reluctance to follow treatment. In addition, insulin injections may be associated with local adverse effects, such as skin necrosis, fat depositions at the sites of injections and infections. It is noteworthy that injectable insulin has a different effect than endogenous insulin. Endogenous insulin is secreted by the pancreas and then enters the portal vein system reaching the liver. In the liver, up to 80% of the endogenous insulin is contained, while the remaining enters the systemic circulation. Thus, a portal systemic gradient of insulin is formed, which controls the levels of insulin in the peripheral tissues, such as the muscles, the adipose tissue and the kidneys [21,22].

In sharp contrast, parenteral exogenous insulin does not undergo this liver entrapment, a fact that results in peripheral hyperinsulinemia. In order to overcome this peripheral hyperinsulinemia, other routes of administration of insulin are being developed, such as the oral route [23]. Insulin administered orally is absorbed by the GI tract and by this intestinal absorption, it enters the portal vein, following the same route as the endogenous insulin. Therefore, as the oral administration of insulin follows the same destination as the endogenously secreted insulin, peripheral hyperinsulinemia is avoided. Moreover, the oral administration of insulin is much more convenient for the patient. However, orally administered insulin faces barriers due to the degradation in the acidic stomach environment, the degradation by enzymes as well as the decreased intake by the intestinal cells due to the presence of mucus and mainly the tight junctions. These major drawbacks could be overcome by the use of various nano-formulas.

In particular, NPs are intended to resist the highly acidic gastric pH as well as to further increase intestinal permeability. The mucus layer together with the tight junctions between the enterocytes form an especially difficult intestinal barrier for the entry of orally administered insulin into the enterocytes [24,25,26,27]. Due to the aforementioned reasons, chitosan-based (CS) NPs have been extensively studied. CS-based NPs have the potential to form electrostatic bonds with the anionic background of mucin due to their cationic features [28]. In addition, they have the capacity to interfere with tight junctions and weaken their permeability. In particular, their interactions with junctional adhesion molecule-1 (JAM-1), claudin-4 and zona-occludens-1 are suggested to result in enhanced intestinal permeability [28,29]. Thus, CS-based NPs have been suggested to facilitate increased entry into enterocytes when compared to other formulations. The CS-based NPs exhibit adhesiveness, cellular penetration, biocompatibility and low toxicity [29,30]. Notably, this increased intestinal permeability due to interfering with tight junctions may also be accompanied by intrusion of pathogenic microorganisms of the gut microbiota via the intestinal barrier. Therefore, despite the fact that CS-based NPs are associated with enhanced intestinal permeability and thus better cellular intestinal penetration, this could lead to the phenomenon of the “leaky gut” and to dysbiosis [31]. “Leaky gut” refers to impaired intestinal barrier, which allows for the intrusion of pathogenic bacteria from the gut lumen to the systemic circulation, leading to a state of endotoxemia [32]. Notably, increased intestinal permeability may permit, apart from the increased cellular penetration of NPs, the intrusion of bacteria and their lipopolysaccharide (LPS) in the systemic circulation, with unknown results [31,32].

In addition, CS-based NPs show enhanced solubility at the low gastric pH, which results in earlier release of insulin in the stomach. This drawback, which restricts the controllable manner of insulin release, has been overcome by the ionic gelation method. More specifically, dextran sulfate (DS), which is a negatively charged anionic polymer, has been used together with the cationic CS-based NPs [33,34]. Pecheckin et al. developed a chitosan–dextran sulfate (CS-DS) nanoformulation for the oral delivery of insulin [35]. More recently, Fathy et al. introduced silica-coated CS-DS NPs and compared their performance to uncoated CS-DS NPs. Their findings demonstrated that the silica-coated NPs exhibited improved release characteristics for orally administered insulin across various pH environments. They concluded that these silica-coated CS-DS NPs warrant further investigation, as their controlled release behavior under different pH conditions suggests strong potential for addressing the challenges associated with oral protein delivery systems [36].

Apart from chitosan-based NPs, other preparations, such as sodium alginate, hyaluronic acid and synthetic polymers like PLA and PLGA, are currently being studied. Polysaccharide-based NPs have also been proposed to be associated with improved biocompatibility that together with their indigenous capacity for substitutions in specific groups make them promising surrogates for oral insulin nanotechnology delivering systems [37,38,39]. Nevertheless, there is still the issue of limited absorption of orally administered polysaccharide-based NPs. When compared to polysaccharide-based NPs, solid lipid NPs due to their lipid components allow for the protection of proteins by degrading enzymes, namely proteases, trypsin, chymotrypsin and pepsin in the GI tract, while providing less toxicity [34,40]. There are various solid lipid NP formulations currently being studied. These solid lipid NPs are more frequently based on fatty acids, such as palmitic acid and stearic acid, and partially on glycerides, such as glyceryl palmitostearate and glyceryl monostearate, and triglycerides [40]. The lipid nature of the aforementioned NPs accounts for their promising potential as a nano-formula for orally administered insulin. Nevertheless, they also have cons, like their short circulation time together with a low encapsulation ability for the time being [20,41]. The same holds true for liposomes, which are widely used due to their high biocompatibility and excellent safety profile, but they also exhibit a low encapsulation efficacy [20,41].

It is noteworthy that Eudragit, which is an NP made of polymers of methacrylic acid esters, is very promising as it enhances the absorption of insulin by Peyer’s patches in the intestines. By combining Eudragit RS (polymer of methyl methacrylate, ethyl acrylate and methacrylic acid ester with ammonium groups) with poly-ε-caprolactone (PCL), there is synergistic intestinal absorption of insulin by the M cells of the Peyer’s patches in the ileum [21]. Other promising options include the combination of organic/inorganic material into nanocapsules for oral insulin delivery. Inorganic compounds are more stable and may lead to better drug protection, when compared with organic materials [21].

On the other hand, organic compounds may improve the functionality of the nano-formula [42]. In particular, there are common organic/inorganic nano-formulas used, like the mesoporous silica NPs and hydroxyapatite NPs. Mesoporous silica NPs have the advantage of a modifiable porous size and outer membrane, while they also exhibit very good biocompatibility [42]. Zhang et al. had the idea to introduce a membrane penetrating peptide on the surface of a mesoporous silica NP, thus mimicking viruses. These NPs that were mimicking viruses in endocytosis by the enterocytes were documented to reduce glucose and provide an effective orally administered insulin delivery system [42]. However, the combination of organic and inorganic materials remains highly demanding and challenging, and merits further investigation, given its significant potential [42,43,44]. Table 1 depicts examples of NPs for oral insulin delivery.

Apart from the orally administered insulin delivery systems, other forms of NPs are also being studied. Amongst them, the transdermal administration of insulin has been proposed. Alkrad et al. have incorporated insulin as part of a non-ionic colloid delivery system at the nanoscale level [45]. This nanosystem, which has been developed with an enhancer of penetration dimethyl-sulfoxide (DMSO), allows for the delayed and repeated release of insulin by avoiding multiple injections [45]. However, this transdermal insulin delivery system has a long way ahead. Regarding the nasal and pulmonary routes of insulin administration, although they are already available, their use is restricted due to the unknown bioavailability and disappointing efficacy thus far [21].

### 3.2. Other Antidiabetic Agents

Metformin still remains the drug of choice for the treatment of T2DM. However, it has been associated with GI adverse effects that may limit its use throughout lifetime. Alginate–gelatin NPs carrying metformin hydrochloride have been demonstrated to increase compliance among patients with T2DM [46]. In addition, Cesur et al. have documented that NPs using monodisperse polymer-based materials have resulted in a more controllable release of metformin, avoiding the development of crystals and ensuring a better antidiabetic effect with less adverse effects [47]. Furthermore, polyethylene glycol solid lipid NPs loaded with metformin hydrochloride have been shown to exhibit better anti-diabetic control after 24 h when compared to metformin in its current forms [48]. It is noteworthy that metformin iron-oxide-based NPs have been found to exhibit not only antidiabetic effects but also anti-inflammatory and antioxidant potential. More specifically, these iron-oxide-based NPs enhance endothelial nitric oxide synthase (eNOS) phosphorylation, thereby ameliorating endothelial dysfunction. Consequently, these NPs could have a significant impact on improving cardiovascular outcomes in patients with T2DM [49]. Interestingly, NPs carrying metformin together with curcumin or other phytochemicals have also been developed [50].

Thiazolidinediones and meglitinides have been used in various NP formulations. In particular, pioglitazone nanostructured lipid-based carriers have been demonstrated to exhibit improved release, when compared to pioglitazone used in its current forms [51]. There are also chitosan-based NPs with pioglitazone and curcumin co-delivery systems, which show prolonged and better antidiabetic properties [52]. Notably, the development of nanomaterials loaded with pioglitazone has gained significant research interest in recent years, not only in the context of DM but also its comorbidities, such as stroke and obesity [53]. Furthermore, repaglinide has been developed in a nanoemulsion formulation, which has a more favorable oral bioavailability than the current form of repaglinide [54].

Regarding sodium-glucose co-transporter 2 (SGLT-2) inhibitors, various NP formulations have been investigated to enhance their bioavailability [55]. In particular, nanotechnology-based delivery systems for SGLT-2 inhibitors are under extensive study, owing to their potential for improved site-specific targeting. For instance, You et al. developed mesoporous silica NPs loaded with dapagliflozin, which selectively targeted cardiomyocytes. This approach facilitated the repair of injured cardiomyocytes and promoted cardiac remodeling [56]. Additionally, a dapagliflozin-enriched nanocarrier, formulated as a nano eye-drop, has been shown to improve the drug’s aqueous solubility, thereby enhancing its therapeutic efficacy in the treatment of diabetic cataract. This formulation effectively reduced the expression of aldose reductase (AKR1B1), subsequently decreasing sorbitol accumulation and mitigating oxidative stress. Furthermore, it downregulated the receptor for advanced glycation end-products (RAGE), thereby suppressing inflammation and epithelial–mesenchymal transition (EMT), ultimately preserving lens integrity [57]. In another study, Al-Tantawy et al. formulated gold NPs encapsulating dapagliflozin for the selective targeting of renal tissue in animal models of diabetic nephropathy. Their findings demonstrated that these gold NPs exhibited nephroprotective effects, including the attenuation of renal fibrosis, as evidenced by reduced levels of transforming growth factor-beta 1 (TGF-β1) and matrix metalloproteinase-2 (MMP-2). Moreover, these gold NPs inhibited apoptosis, as indicated by elevated renal expression of the anti-apoptotic gene *Bcl-2*, and promoted autophagy, as reflected by increased expression of Beclin-1 [58].

Injectable glucagon-like peptide-1 (GLP-1) analogs are also well-known for their pleiotropic therapeutic effects. However, their clinical utility is limited by the requirement for subcutaneous administration. Among them, liraglutide requires once-daily subcutaneous injection, a drawback that may potentially be addressed through advances in nanotechnology. In particular, Kweon et al. investigated a nanoformulation of liraglutide designed for oral administration, which demonstrated promising results [59]. Similarly, Subedi et al. developed an oral micelle-based nanoformulation of liraglutide, which exhibited both antidiabetic and lipid-lowering effects in an animal model of DM [60]. Exanetide has also been studied in a chitosan-based NP, which allowed for the oral delivery of exanetide instead of its subcutaneous administration. This oral NP has been shown to be promising due to the increased endocytosis by the enterocytes and the easier penetration of the mucus in the intestines [61]. As for semaglutide, which is available in both injectable and oral formulations, Pinto et al. investigated a PLGA/PEG NP delivery system. Their findings demonstrated that this PLGA/PEG NP, designed to target the intestinal Fc receptor, significantly enhanced the cellular uptake of semaglutide by enterocytes [62].

Overall, there is ongoing research regarding improvements of non-insulin-based antidiabetic therapies in the light of nanomedicine. Nevertheless, the narrative approach of this review may overlook contradictory evidence. Therefore, future systematic reviews are more than welcome in this context.

Table 2 refers to the pros and cons of nanotechnology in DM, when compared to standard treatment.

**Figure 2:**
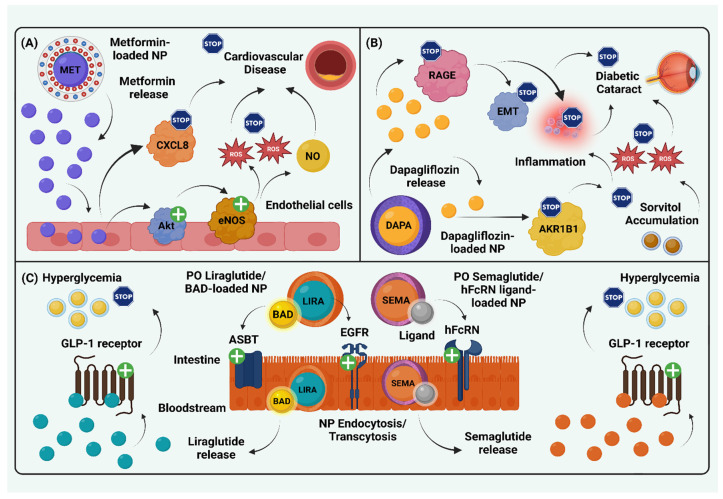
Nanomedicine strategies for targeted drug delivery in diabetes mellitus. (**A**) A nano-pharmaceutical formulation enriched with metformin enabled controlled drug release, leading to a reduction in CXCL8 levels in endothelial cells. Concurrently, metformin activated Akt, which in turn stimulated eNOS, resulting in NO production and attenuation of oxidative stress. Overall, endothelial function was preserved, offering cardiovascular benefits [49]. (**B**) A dapagliflozin-enriched nanocarrier, when administered as a nano eye-drop, enhanced the aqueous solubility of dapagliflozin, increasing its efficacy in diabetic cataract treatment. Dapagliflozin reduced AKR1B1 expression, leading to decreased sorbitol accumulation and mitigation of oxidative stress. Additionally, it downregulated RAGE, thereby attenuating both inflammation and EMT, ultimately preserving lens integrity [57]. (**C**) A nanoparticle loaded with liraglutide, and BADs interacted with ASBT on the surface of intestinal epithelial cells. Upon nanoparticle binding to ASBT, bile acids were recognized and transported intracellularly. Simultaneously, liraglutide, incorporated within the nanoparticle, activated EGFR, promoting endocytosis and transcytosis. The nanoparticle traversed the intestinal epithelium and entered systemic circulation, releasing liraglutide, which subsequently acted on GLP-1 receptors to regulate blood glucose levels [60]. Similarly, oral semaglutide was encapsulated in a nanoparticle surface-modified with either a peptide or an affibody targeting hFcRn. The nanoparticle was bound to hFcRn on enterocyte surfaces, facilitating endocytosis and transcytosis across the intestinal barrier. This mechanism enhanced gastrointestinal stability and absorption of semaglutide, which otherwise exhibits low permeability due to its high molecular weight, thereby ensuring its anti-hyperglycemic effect through GLP-1 receptor activation [62]. Abbreviations: AKR1B1: aldose reductase 1B1; ASBT: apical sodium-dependent bile acid transporter; BAD: bile acid derivative; CXCL8: C-X-C motif chemokine ligand 8; DAPA: dapagliflozin; eNOS: endothelial nitric oxide synthase; EGFR: epidermal growth factor receptor; EMT: epithelial–mesenchymal transition; GLP-1: glucagon-like peptide-1; hFcRn: human neonatal Fc receptor; LIRA: liraglutide; MET: metformin; NO: nitric oxide; NP: nanoparticle; RAGE: receptor for advanced glycation end-products; PO: per oral; SEMA: semaglutide; +: activation; →: leads. Created in BioRender. Kounatidis, D. (2025) https://BioRender.com/m12t721. Assessed on 16 March 2025.

Figure 2 illustrates distinct nanotechnology-based drug delivery systems for the treatment of DM and its complications.

## 4. Phytomedicines with Antidiabetic Properties and Nanotechnology

Phytochemicals (PHYs) are bioactive compounds that are plant-derived. It is widely known that certain PHYs exhibit powerful antidiabetic properties. However, their antidiabetic potential is restricted due to their poor bioavailability, as most of them are insoluble in water. In addition, the vast majority of PHYs are degraded by enzymes as well as the gut microbiota before reaching their intended site of action. In addition, their short elimination time further limits their therapeutic potential. By applying nanotechnology to PHYs, these obstacles may be overcome [63,64,65,66]. In fact, PHYs such as curcumin, resveratrol, berberine, silymarin and anthocyanins are among the most well-known PHYs that exert antidiabetic features. Table 3 describes major PHYs, which are currently being investigated as potential antidiabetic agents with nanoformulation-based drug delivery systems. Notably, out of the 25 references in Table 3, 13 refer to animal models, 8 are in vitro studies and 4 are review articles. This lack of clinical studies is suggestive of the very early phase of PHYs in the setting of nanotechnology.

## 5. Gene Therapy and Nanomedicine in Diabetes Mellitus

Nowadays, gene therapy aims to add a gene that is missing or insert DNA, RNA or small interfering RNA (siRNA) in order to enhance or mitigate the function of defective genes. In DM, there is ongoing research in terms of gene therapy for insulin production as well as for genetic loci implicated in the progression of T1DM and T2DM. Notably, approximately 75 genetic loci have been demonstrated to be involved in T2DM progression and could therefore be exploited as therapeutic targets [93]. For instance, NPs with the polymer polyethylene imine have been shown to increase the production of insulin by pancreatic cells by carrying the gene responsible for the GLP-1 receptor [94]. Apart from the application of gene therapy in improving insulin production directly from pancreatic cells, there are other potential candidates, such as silencing glucagon receptors by using siRNAs. So far, lipid NPs have been developed to restore glucose homeostasis by increasing plasma levels of glucagon [95]. Moreover, in patients with T2DM receiving metformin, genetic variations in the solute carrier family 22 member 1 (*SLC22A1*) loci have recently been associated with differential therapeutic responses and variability in glycated hemoglobin (HbA1c) levels [96].

Inflammation-related genes in patients with T2DM have also been a subject of investigation. For example, genetic loci that inhibit the expression of the nucleotide-binding oligomerization domain-like receptor protein 3 (NLRP3) inflammasome have been identified. These loci have been shown to mitigate inflammation and delay the progression to T2DM in animal models by interfering with NLRP3 inflammasome activation [97]. In addition, in T1DM, insulitis has been proposed as a key pathogenetic mechanism underlying disease development. In this context, gene therapy strategies aimed at reducing pancreatic inflammation have been explored, including the use of CS-based NPs for the targeted delivery of a plasmid encoding interleukin-4 (IL-4) and interleukin-10 (IL-10). Ko et al., in their study with CS-based NPs, have documented regression of insulitis and autoimmunity in animal models of T1DM [98]. In another study, poly-α-4-aminobutyl-1 NPs carrying a plasmid encoding IL-10 were administered to non-obese diabetic (NOD) rodents. This nanoformulation led to a significant reduction in insulitis and, consequently, regression of T1DM in this animal model [99]. Despite the promising outcomes demonstrated in preclinical studies, gene therapy approaches employing NPs for both T1DM and T2DM remain far from being established as standard treatment modalities. Nevertheless, ongoing advancements in NP-based gene therapy are highly anticipated and hold considerable potential for future clinical application.

## 6. Nanomedicine for Transplantation of Pancreatic Cells for Diabetes Mellitus Management

Conventional methods for the transplantation of pancreatic cells have been associated with significant adverse effects, primarily due to the necessity of immunosuppressive therapy and the complex procedures involved in isolating pancreatic islets from suitable donors [100,101,102,103,104,105,106,107,108,109]. In this context, nanomedicine offers promising alternatives for simplifying and improving the isolation of stem cells, which could play a pivotal role in the treatment of DM. Specifically, current approaches utilize technologies such as Clustered Regularly Interspaced Short Palindromic Repeats/CRISPR-associated protein 9 (CRISPR/Cas9) or the transfection of stem cells with plasmids to enhance gene editing. These edited genes are often encapsulated in lecithin or cationic lipid-assisted PEG/PLGA NPs. This approach helps to mitigate host–foreign body responses and protects the transplanted cells from immune rejection, thereby ensuring insulin homeostasis [100,101,102,103,104,105,106,107,108,109].

## 7. Nanotechnology and Diabetes Treatment: Local Applications for Diabetic Wound Healing

Nanotechnology in the treatment of DM has not only focused on enhancing systemic antidiabetic therapies but has also made significant strides in diabetic wound healing. In this context, there is particular interest in nitric oxide (NO)-based nano dressings, as NO is well-known for its wound-healing properties [110,111,112,113]. Additionally, hypoxia-inducible factor 1-alpha (HIF-1α) plays a crucial role in promoting angiogenesis and tissue restoration due to its beneficial effects on cellular responses to hypoxia. However, HIF-1α dysfunctions in patients with DM and diabetic wounds are caused by the presence of degrading enzymes. Consequently, the exogenous administration of HIF-1α at the wound site has been proposed as a potential therapeutic strategy to enhance diabetic wound healing [114,115,116]. Furthermore, the incorporation of antimicrobial substances into nanogels is under investigation, with growing global interest in their application. For example, NPs such as silver NPs have been extensively studied, although further research is needed to assess their efficacy and potential toxicity [117,118]. Given these considerations, localized nanotechnology applications, particularly the use of nanomaterials directly at the site of the wound, are of paramount importance [113,114,115,116,117,118,119]. Indeed, the field of localized nanotherapy for diabetic wound healing is expanding rapidly, further accelerated by the integration of artificial intelligence (AI), which enables the exploration of not only chemical compounds but also phytochemicals in therapeutic approaches [120,121].

## 8. Conclusions

In conclusion, nanomedicine’s contribution to the treatment of T1DM as well as T2DM seems to be very promising. Nevertheless, a variety of NPs are eagerly anticipated in order to enrich our armamentarium regarding DM therapy. Nowadays, nanomedicine is rapidly evolving with the use of high-throughput strategies and computer-assisted learning. The ability to design NP libraries and the use of AI for further selecting the most appropriate and compatible NPs would pave the way for revolutionizing DM treatment. However, the high costs together with uncertainties regarding future adverse effects are some of the current limitations of the widespread use of nanotechnology in the management of DM. As the exact interactions of NPs with the human body still remain poorly understood, their safety in the long term should be further explore. In addition, an issue is the timeline for NPs to enter clinical trials, especially Phase 3 studies, in order to be fully implemented into clinical practice. Therefore, the aforementioned caveats should not be overlooked as it seems likely that nanotechnology in the management of DM has a long way ahead.

## Figures and Tables

**Table 1:** Examples of NPs being studied for the oral administration of insulin.

Material	Carrier Compound	Method Used	Size (nm)
Chitosan	Chitosan, alginate	Electrostatic bonds and chemical interactions	104
Chitosan	Chitosan	Self-assembly	277
Chitosan	Carboxymethyl chitosan	Chemical cross linking interactions	190
Chitosan	Chitosan, γ-PGA	Electrostatic bonds	250
	Alginate, dextran sulfate	Emulsification/gelation	300
	HPMCP	Emulsification/solvent diffusion	200
	Proanthocyanidines, glucans	Recrystallization	100–300
PLA	PLA/PEG	Nanoprecipitation	63
PLGA	PLGA 20 kDa/50 kDa	Double emulsion	157/247
MOFs	Fe-based MOF	Physical absorption	100
	Zr6-based MOF	Physical absorption	(-)
DOTAP, EPC	BSA	Thin-film hydration	195
EP, CH, DOTAP	Chitosan	Thin-film hydration	439
DODA-501, NIPAAm, AAC		Free radical polymerization	94–200
Soybean lecithin	Peptide	Double emulsion	161.6
Soy lecithin	Propylene glycol	Emulsification/solvent evaporation	203.6
Hyaluronic Acid, HPMCP	Penetratin peptide	FNC	103
Mesoporous silica NPs	KLPVM peptide	Physical absorption	263.5
Hydroxyapatite, PEG	Gallic acid	Homogeneous Precipitation/esterification/amidation	150
Mesoporous silica NPs	APBA	Aqueous polymerization/physical absorption	202.8

Abbreviations: AAC: acrylic acid; APBA: 2-aminophenylboronic acid; BSA: bovine serum albumin; CH: cholesterol; DOTAP: N-1-2,3 dioleoyloxy propyl-N-N-N-trimethyl ammonium methylSulfate; EPC: egg phosphatidyl-choline; Fe: ferrum; FNC: flash nano-complexation; HPMCP: hydroxypropyl-methylcellulose phthalate; MOFs: metal–organic frameworks; NIPAAm: N-isopropylacrylamide: PEG: polyethylene glycol; PGLA: poly lactic-co-glycolic acid; PLA: polylactic acid; Zr: zirconium.

**Table 2:** Pros and cons of nanotechnology in diabetes mellitus management.

Pros of NPs	Cons of NPs
Improved absorption	Unknown safety in the long term
More controllable release allowing for plausible better compliance	Lack of clinical trials
Resistance to various pH values as well as to enzymatic degradation throughout the GIT	High costs
Improved entry into the targeted cells	Research is in its very early stages

**Table 3:** Main natural compounds with antidiabetic features explored with nanoformulation-based drug delivery systems.

PHYs.	Antidiabetic Properties/Action
Curcumin	↓ FPG; ↓ IR Also used in diabetic wounds in a nanoformula hydrogel as it has healing properties due to its inhibition of MMP-9 [67,68].
Resveratrol	↓ FPG; ↓ IR It is undergoing evaluation on the treatment of DR due to its inhibition of VEGF-1,ICAM-1, MCP-1 and ERK1/2 [69,70].
Berberine	↓ FPG; ↓ IR [71].
Silymarin	↓ FPG; ↓ IR [72].
Naringenin	Under investigation for improvement in early DR due to its antioxidant properties. Amelioration in DKD due to inhibition of ferroptosis via the SIRT1/FOXO3a pathway [73,74,75].
Quercetin	It may be useful in DR, DKD and DN due to its antioxidant, anti-fibrotic, anti-inflammatory potential and by affecting pyroptosis. As a hydrogel, it is postulated to improve wound healing due to its antioxidant properties [76,77].
Rosmarinic Acid	It has been suggested to ameliorate cardiac dysfunction (cardiomyopathy) in DM due to its antioxidant properties. Instillation on the eyes has been proposed to improve DR due to its antioxidant capacity. Also, it is undergoing evaluation as a gel for diabetic wounds. In addition, it has been suggested to interfere with the deposition of β-amyloid in the brain [78,79,80,81].
Thymoquinone (from *Nigella sativa*)	It is suggested to possess nephroprotective potential via the Nrf2/NOX2 pathway. It has been suggested to be useful in diabetic wounds due to its antioxidant, anti-inflammatory and antimicrobial properties as well as its angiogenesis amelioration [82,83,84,85,86].
Ferulic Acid	It has been implicated in ameliorating DKD by means of improving autophagy. It has been suggested as a nanogel to be involved in healing diabetic wounds due to its antioxidant and antimicrobial potential [87,88,89].
Seagrass *Halodule uninervis*	Very recently, it has been suggested to exhibit antioxidant and anti-inflammatory properties [90].
*Arbutus unedo*	It has been proposed to exert antidiabetic, antioxidant, anti-inflammatory as well as antimicrobial potential [91].
Epigallocatechin-3 gallate	This polyphenolic compound of tea has been suggested to inhibit angiogenesis in the eye by targeting integrins; as such, it may be further exploited in DR [92].

↓:reduction. Abbreviations: DN: diabetic neuropathy; DKD: diabetic kidney disease; DR: diabetic retinopathy; ERK1/2: extracellular signal-regulated kinase 1/2; FPG: fasting plasma glucose; ICAM-1: intercellular adhesion molecule-1; IR: insulin resistance; MCP-1: monocyte chemotactic proteins-1; MMP-9: matrix metallo-proteinase-9; VEGF-1: vascular endothelial growth factor-1.

## Data Availability

Not applicable.

## Abbreviations

AAC: acrylic acid; AI: artificial intelligence; AKR1B1: aldose reductase 1B1; APBA: 2-aminophenylboronic acid; ASBT: apical sodium-dependent bile acid transporter; BAD: bile acid derivative; BSA: bovine serum albumin; CH: cholesterol; CRISPR/Cas9: Clustered Regularly Interspaced Short Palindromic Repeats/CRISPR-associated protein 9; CS: Chitosan; CXCL8: C-X-C motif chemokine ligand 8; DAPA: dapagliflozin; DKD: diabetic kidney disease; DM: diabetes mellitus; DMSO: dimethyl-sulfoxide; DOTAP: N-1-2,3 dioleoyloxy propyl-N-N-N-trimethyl ammonium methylSulfate; DR: diabetic retinopathy; DS: dextran sulfate; eNOS: endothelial nitric oxide synthase; EGFR: epidermal growth factor receptor; EMT: epithelial–mesenchymal transition; EPC: egg phosphatidyl-choline; ERK1/2: extracellular signal-regulated kinase 1/2; Fe: ferrum; FNC: flash nano-complexation; FPG: fasting plasma glucose; GLP-1: glucagon like peptide-1; GI: gastrointestinal; HbA1c: glycated hemoglobin; hFcRn: human neonatal Fc receptor; HIF-1a: hypoxia inducible factor-1a; HPMCP: hydroxypropyl-methylcellulose phthalate; ICAM-1: intercellular adhesion molecule-1; IL: interleukin; IR: insulin resistance; LIRA: liraglutide; LPS: lipopolysaccharide; MCP-1: monocyte chemotactic proteins-1; MET: metformin; MMP-9: matrix metallo-proteinase-9; MOFs: metal–organic frameworks; NIPAAm: N-isopropylacrylamide: PEG: polyethylene glycol; NLRP3: nucleotide-binding oligomerization domain-like protein 3; NO: nitric oxide; NOD: non-obese diabetic; NP: nanoparticle; PEG: polyethylene glycol; PGLA: poly-lactic-co- glycolic acid; PLA: polyLactic acid; PHY: phytochemical; RAGE: receptor for advanced glycation end-products; SEMA: semaglutide; SGLT-2: sodium-glucose co-transporter 2; siRNA: small interfering RNA; T1DM: type 1 diabetes mellitus; T2DM: type 2 diabetes mellitus; TGF-β1: transforming growth factor-beta 1; VEGF-1: vascular endothelial growth factor-1; WHO: World Health Organization. Zr: zirconium.
